# Environmental Consciousness and Organic Food Purchase Intention: A Moderated Mediation Model of Perceived Food Quality and Price Sensitivity

**DOI:** 10.3390/ijerph17030850

**Published:** 2020-01-29

**Authors:** Jianming Wang, Thuy Linh Pham, Van Thac Dang

**Affiliations:** 1 School of Business Administration, Zhejiang University of Finance & Economics, Hangzhou 310018, China; sjwjm@zufe.edu.cn; 2 Department of Business Administration, Business School, Shantou University, Shantou, Guangdong 515063, China; phamlinhhc@gmail.com

**Keywords:** environmental consciousness, perceived food quality, price sensitivity, purchase intention

## Abstract

As purchase power and consumption knowledge increase, consumers gradually demand safer and healthier products. In addition, consumers focus greater attention on organic food. This study investigates the relationship between environmental consciousness and organic food purchase intention with the mediating roles of perceived food quality and price sensitivity. The objective is to shed new light on our understanding of consumers’ perceptions and behavioral intentions toward organic food. Using sample data of 518 consumers in different food retailers in China, empirical results show that environmental consciousness has a positive impact on organic food purchase intention. Perceived food quality has a mediating effect on the link between environmental consciousness and organic food purchase intention. Price sensitivity moderates the relationship between perceived food quality and organic food purchase intention. Furthermore, price sensitivity moderates the indirect effect of environmental consciousness on organic food purchase intention through perceived food quality.

## 1. Introduction

Green purchase behavior has been a widely discussed topic in recent decades [1]. Economic development, competition, and an explosion of market information have increased consumers’ purchasing power and consumption knowledge [2]. For example, consumers can obtain various product information due to the development of digital technology and the Internet. Competing alternatives also offer numerous product options for consumers [2]. Hence, consumers gradually demand better quality and less environmentally harmful products [3]. Green and healthy products have become the first choice for consumers in today’s environment [4].

Organic food is often viewed as healthy products that provide more nutrition and less harm to human health [4]. As the market for organic food increases rapidly, knowledge about consumer behavior regarding the consumption of organic food will provide implications for researchers and business managers to construct better decisions and policies [5]. Therefore, the need for understanding consumers’ perceptions and behavioral intentions toward organic food becomes critically important.

Grimmer and Miles [6] and Mishal et al. [1] suggested that knowledge and perceptions about environmental issues may affect consumers’ purchase intention regarding green products. Consumers who care more about environmental and ethical issues prefer purchasing environmentally friendly products [4]. Given the substantial research on organic food in previous literature, the effect of environmental consciousness on organic food purchase intention has been underdetermined. Furthermore, research on organic food focusing on the relationship between mediating and moderating mechanisms is limited, thus also limiting the knowledge to the current literature. In addition, in emerging markets (i.e., China), consumer purchasing power and consumption knowledge gradually increase [2]. As consumers demand more environmentally friendly and organic food products, the market potential for green products also increases. However, as compared with developed economies, consumers in emerging markets tend to place significant weight on product quality and price in their purchase decisions [7]. Several studies have also suggested the importance of product quality and price on consumers’ decision-making. For example, Solomon [8] argued that consumers often evaluate products and differentiate products in terms of product quality and price. Hwang and Chung [9] and Suciu, Ferrari, and Trevisan [10] noted that product quality and price are the two most important factors that distinguish organic and conventional foods. Consumers’ perceptions of quality and price may also affect their purchase behavior. Unfortunately, very limited research has determined the mediating and moderating mechanisms of product quality and price sensitivity in the link between consumers’ perceptions and behavior. Specifically, research on the role of perceived food quality and price sensitivity in the link between environmental consciousness and organic food purchase intention is limited. Therefore, to fill these gaps in the previous literature on organic food, this study investigates the relationship between environmental consciousness and organic food purchase intention with a moderated mediation model of perceived food quality and price sensitivity. Ultimately, this study determines the impact of environmental consciousness on organic food purchase intention with the mediating role of perceived food quality. In addition, this study investigates the moderating role of price sensitivity on the link between perceived food quality and organic food purchase intention. Moreover, this study examines the moderating role of price sensitivity on the indirect effect of environmental consciousness on organic food purchase intention through perceived food quality.

This study contributes to the current literature on organic food in several manners. The findings of this study will help clarify the relationship between environmental consciousness and organic food purchase intention. Knowledge about this relationship helps to fill the research gap in previous literature that has provided very limited clues about this relationship. Furthermore, this study extends the current literature on organic food by providing empirical evidence to explain the mediating and moderating mechanisms of perceived food quality and price sensitivity into the link between environmental consciousness and organic food purchase intention. Thus, this study clarifies not only the direct effect but also the indirect influence of environmental consciousness on organic food purchase intention. In addition, this study provides important implications for practical managers in the food industry to understand consumer behavior and construct better decisions to satisfy consumers’ needs about organic foods. Moreover, this study utilizes Chinese consumers as the sample data to determine the issue of organic food consumption. The findings of this study will provide implications for managers of domestic and foreign food companies to make better decisions and policies in China’s food market.

The structure of this paper is organized as follows. Theoretical background and hypotheses are discussed in Section 2. Sample data and measures are explained in Section 3. Empirical results are demonstrated in Section 4. Discussion and conclusions are discussed in Section 5 and Section 6.

## 2. Theoretical Background and Hypotheses

### 2.1. Environmental Consciousness

In recent decades, researchers and practitioners have focused on environmental issues. Stern et al. [11] proposed value–belief–norm theory of movement support to explain the phenomenon of the environmental movement. The authors identified five variables, namely, values, new ecological paradigm, awareness of consequences beliefs, ascription of responsibility beliefs, and personal norm for pro-environmental action. The causal chain shifts from values and perceptions to more focused beliefs about human–environmental relations, finally activating a sense of moral obligation that creates actions toward environmental goals. Dunlap et al. [12] developed a new measurement scale to revise the New Environmental Paradigm (NEP) scale, which was designed to measure pro-environmental orientation. This new scale consists of 15 items and has high internal consistency, reliability, and validity. The revised NEP scale was widely used in previous literature to measure environmental behavior [13,14,15]. Keszey [16] conducted research with 296 firms in Hungary to determine the environmental orientation of business firms. He adopted a measurement scale with five items to measure employees’ perceptions about the importance of environmental issues to their firms. Prakash, Singh, and Yadav [17] also developed a measurement scale to measure consumers’ level of consciousness with environmental issues. This measurement scale clearly indicates consumers’ perceptions and their willingness to solve environmental problems regarding their purchase behaviors. Prakash et al.’s [17] scale measures consumers’ environmental consciousness in the context of retailers. This scale was adopted in the present study because it is suitable for the context of consumers who purchase organic food products in physical retailers. In this study, environmental consciousness reflects the degree to which consumers care about environmental issues (i.e., environmentally friendly consumption behavior) [5]. Environmental consciousness refers to “psychological factors that determine consumers’ propensity towards pro-environmental behaviors” [1].

### 2.2. Organic Food

Organic food is more environmentally friendly than conventional food. It also is more nutritious contains less harmful ingredients, and is of higher quality than conventional food [7,10,18]. Organic food often requires strict regulations and rules for production, distribution, and preservation and is naturally produced without chemicals [9,19]. Given that organic food is viewed as healthier and safer for human health and the environment, consumers often hold positive attitudes and behaviors toward organic food [10,18,20]. Consumers often evaluate organic food in terms of nutrition, taste, freshness, quality, and price [7,19,21]. The environmental and ethical concern is also an important factor that may affect consumers’ perceptions of organic food [5].

Previous literature has placed great efforts in exploring consumer motivation to purchase organic food products [1,3,4,5,22]. Several antecedents of organic food purchase behavior have been identified in previous studies. For example, Shamsi, Najafabadi, and Hosseini [23] determined the relationship between consumer attitudes and organic food consumption behavior. Sultan et al. [24] also examined the effect of consumer attitudes on purchase behavior. Boobalan and Nachimuthu [7] reported the predictive ability of consumer attitudes, responsive efficacy, and perceived behavioral control on purchase intention. Molinillo, Vidal-Branco, and Japutra [19] found that health and social consciousness have positive associations with purchase behavior. Chekima et al. [4] suggested that consumer attitudes, subjective norms, and perceived availability are the main predictors of organic food consumption. Hwang and Chung [9] reported that store quality, price perception, and corporate social responsibility beliefs are the main antecedents of purchase behavior. In a reviewing research, Kushwah et al. [18] summarized five main factors as motives for organic food consumption, namely, functional, social, emotional, conditional, and epistemic values. Similarly, Massey, O’Cass, and Otahal [25] reviewed organic food literature and concluded that most previous studies have used product-related, supply-related, and consumer psychographic and social demographic variables as main antecedents to predict purchase behavior. Scalco et al. [26] also conducted meta-analytic research and concluded that previous literature has widely used planned behavior theory to determine organic food consumption. Predictor variables of planned behavior theory include consumer attitudes, subjective norms, and perceived behavioral control [27,28]. Shamsi et al. [23] and Molinillo et al. [19] suggested that more variables should be explored to provide additional insights on consumers’ perceptions and behaviors toward organic food consumption.

### 2.3. Environmental Consciousness and Organic Food Purchase Intention

Environmental and ecological motives may affect consumers’ attitudes and behaviors toward green products [3]. For example, Hamm and Gronefeld [29] found that consumers tend to purchase environmentally friendly products because these products have less impact on the environment. Zepeda and Deal [30] suggested that consumers often take environmental protection and animal welfare into their decisions when they purchase green products. D’Amico, Di Vita, and Monaco [31] found that consumers are more willing to pay a higher price and purchase more organic wines. These consumers often associate the impact of their purchase behavior with environmental and ecological systems. Leaniz, Crespo, and Lopez [2] reported that consumers choose to stay at environmentally certified hotels because they suppose that these hotels provide better quality and help protect the environment. The findings of these studies suggest that consumers care more about environmental issues and integrate environmental impacts into their purchase decisions [19,22]. Consumers who hold high levels of environmental consciousness care more about environmental protection and its impact on human health and society. These consumers may believe that if they purchase environmentally friendly products, then firms will invest and employ greater efforts in producing environmentally friendly products [32]. Firms will also care more about the environment and display ethical behaviors because they must uphold a good image and satisfy consumers’ needs [19]. Therefore, environmentally conscious consumers may suppose that their green purchase behaviors will have a positive impact on the environment and ecological system. Fundamentally, when consumers care more about environmental issues and possess high consciousness about environmental protection, they will seek to lessen the impact of their behavior on the environment. Consequently, environmentally conscious consumers will purchase more organic food because they perceive that organic food has less impact on the environment than conventional food. Thus, the following hypothesis is developed.

**H1.**  *Environmental consciousness will be positively related to organic food purchase intention.*

### 2.4. Mediating Role of Perceived Food Quality

Product quality refers to the attributes and characteristics of a product. Good quality indicates that the product is reliable and effectively performs its functions. High-quality products also have the ability to satisfy consumers’ needs and create more value for consumers [8,33]. Organic food is often viewed as having higher quality than conventional food because it is naturally produced without pesticides, bioengineering, and synthetic fertilizers [7,19]. Consumers often prefer to purchase organic food due to health benefits, safety, taste, and nutritional value of organic food [6,24]. Product quality is a key factor in consumers’ decision-making [10,19,21]. Quality of organic food is also an important consideration in consumers’ decisions when they purchase food products [23]. When consumers perceived high-quality organic food, they may perceive that organic food is better than conventional food [10]. Consumers also believe that organic food is the best choice for their health and the environment because organic food products are natural and have no harmful elements. Consequently, consumers often choose to purchase organic food when they take the quality of food into their purchase decision [9].

Environmental concern and ecological consideration also affect how individuals perceive and evaluate products [1]. People who care more about the environment often consider the impact of their behavior on the environment. When purchasing products, environmentally conscious people often link products with ethical and environmental issues [22]. For example, environmentally conscious consumers who purchase a car may consider the car’s impact on the environment. They may perceive that electric cars have better quality and produce less pollution. Therefore, these consumers may prefer to purchase electric cars. The logic is also similar in the food market. Consumers who care about the environment and ecological system may possess rich knowledge and tend to learn more about food products because these consumers may consider the relationship between food products and the environment [3]. When considering the environmental impact of food products, consumers may perceive and evaluate organic food as of high quality because organic food is generally known as natural products without chemicals [31]. Ultimately, environmentally conscious consumers may believe that organic food holds higher quality than conventional food because organic food is nutritious, fresher, and safer. The production of organic food also has a less negative impact on the environment. Consequently, consumers may hold a high intention to purchase organic food. Therefore, when consumers care about the environment, they will seek more information and care about food products that can impact the environment. These consumers may perceive organic food as high-quality when compared with conventional food, and consumers will purchase more organic food because it gives them more benefits. That is, environmental consciousness will increase consumers’ perception of the quality of organic food, which in turn enhances consumers’ intention to purchase organic food. The following hypothesis is developed.

**H2.**  *Perceived food quality will mediate the relationship between environmental consciousness and organic food purchase intention.*

### 2.5. Moderating Role of Price Sensitivity

Price sensitivity refers to the degree to which consumers’ purchasing behavior is affected by the price changes of a product [30,34]. High-price sensitivity indicates that a slight change in price will have a substantial effect on consumers’ purchase behavior [33,35]. Price is often a key component of the product positioning of a company. Consumers often view that high price represents high product quality and vice versa. By contrast, low quality is associated with a low price [34,36]. In the food product market, consumers often view organic food as high-price products that offer more benefits for consumers as compared with conventional foods [18]. Price has been reported as a key factor that has a direct influence on consumers’ intention to purchase organic food. For example, Ghali-Zinoubi and Toukabri [37], Prakash et al. [17], and Hwang and Chung [9] found a positive direct relationship between price and consumers’ intention to purchase organic food.

Given that price is a key factor in consumers’ purchase decisions, perceptions about quality and behavioral intention may differ between consumers who are price-sensitive and insensitive. Consumers who are price-sensitive may take price as the key consideration in their purchase decision. These consumers may evaluate organic food quality and decide to purchase depending largely on the price of organic food [18,24]. If the price is inexpensive, they tend to purchase more and believe that they gain more benefits from their purchases [37,38]. However, if the price of organic food is high, price-sensitive consumers may purchase less organic food and more conventional food because they may think that conventional food is also acceptable in product quality [4,5]. The effect of perceived organic food quality on purchase intention is weakened for consumers who are price-sensitive and consider price as a key factor in their purchase decision. By contrast, consumers who are price-insensitive may not care much about the price of organic food. Instead, these consumers tend to evaluate organic food in terms of nonprice factors, such as freshness, nutrition, taste, and safety [7,19]. When price-insensitive consumers believe that the quality of organic food is good, they will purchase more products because they consider only product quality and put less weight on price factors [37,38]. Therefore, the effect of perceived organic food quality on purchase intention will be stronger for consumers who are price insensitive. The following hypothesis is developed.

**H3.**  *Price sensitivity will moderate the relationship between perceived food quality and organic food purchase intention. The relationship is stronger when price sensitivity is low and weaker when price sensitivity is high.*

**Figure 1:**
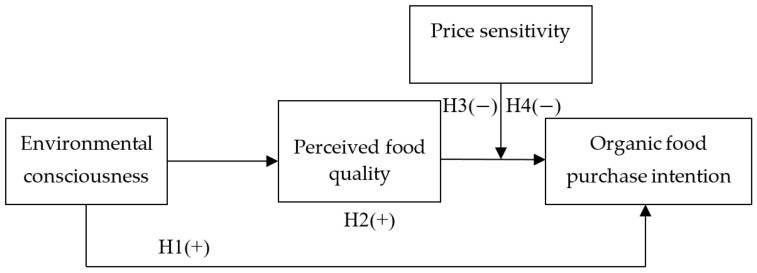
Research framework.

Figure 1 shows a conceptual model of this study. As indicated, perceived food quality mediates the relationship between environmental consciousness and organic food purchase intention. In addition, price sensitivity moderates the link between perceived food quality and organic food purchase intention. Thus, the indirect effect of environmental consciousness on organic food purchase intention by perceived food quality is strong when price sensitivity is low and weak when price sensitivity is high. Accordingly, the following hypothesis is developed.

**H4.**  *Price sensitivity will moderate the indirect effect of environmental consciousness on organic food purchase intention via perceived food quality. The indirect effect is stronger when price sensitivity is low and weaker when price sensitivity is high.*

## 3. Method

### 3.1. Sample and Data Collection

A Chinese version of the questionnaire was designed from a translation from English to Chinese and backward translation from Chinese to English. A pilot test with 50 consumers was conducted to check the clarity of measurement items. After ensuring the quality of the questionnaire, a formal survey was conducted in different food retailers in Guangdong, China. Guangdong is one of the largest provinces with a population of 113.46 million people, which is the largest province by GDP in Mainland China. We adopted a systematic random sampling technique to collect data because the population list was not available. Our research team personally approached consumers when they visited physical food retailers. Each one out of three consumers was randomly invited to participate in the survey. They voluntarily completed the questionnaire with the assistance of our research teams. Given that a probabilistic sampling is extremely difficult in the real world, and in the case that the population list was absent, we used systematic random sampling to enhance the randomness of the sampling procedure [39]. After 3 months (from April to June 2019), this study collected 560 questionnaires; of which, 518 questionnaires were valid with a response rate of 92.5%. Minor samples of 42 questionnaires were excluded from the final data because of missing values.

### 3.2. Sample Characteristics

Table 1 presents the sample characteristics of respondents in this study. Approximately 64.1% of respondents were female and 35.9% were male. Approximately 85.5% of respondents were aged between 26 and 45, only 7.1% were 25 years old or younger, and approximately 7.3% were 46 years old or older. Most respondents were married (96.7%), and a very small portion of respondents was not (3.3%). About 68.0% of the respondents had high school or below education, 29.3% had university or college education, and only 2.7% were graduate or above education. Approximately 26.3% of respondents had income under 3000 RMB, 43.6% had income between 3000 and under 6000 RMB, 18.5% had income between 6000 and under 9000 RMB, and approximately 11.6% had income 9000 RMB or above. Most of the respondents had previously bought organic food (80.7%), and only 19.3% had not previously bought organic food.

### 3.3. Measures

Measurement items in this study were adopted from previous literature. Table 2 shows all the constructs and items in this study. Environmental consciousness was measured with three items adapted from Prakash et al. [17]. This scale measures consumers’ level of consciousness with environmental issues and their efforts and willingness to solve environmental problems. This scale has been widely used in different contexts in marketing and consumer studies [5,17,29,30,31]. Thus, we selected this scale to measure environmental consciousness in this study. The items included “The balance of nature is very delicate and can be easily upset,” “I have switched products for ecological reasons,” and “When I have a choice between two equal products. I purchase the one less harmful to other people and the environment.”

Perceived food quality was measured with three items adapted from Anselmsson, Burt, and Tunca [40]. This scale measures consumers’ overall perceptions about the quality of organic food in physical retailers where they shopped. This scale was also widely used in different contexts in food marketing literature [41,42,43]. The items were “There is a high likelihood that organic food bought at this store will be of extremely high quality,” “When shopping at this store, I expect to see high quality organic food,” and “Overall, this store sells high quality organic food.”

Price sensitivity was measured with two items adapted from Hsu et al. [36]. The original scales of price sensitivity in Hsu et al. [36] include three items. However, we deleted one item due to its overlap with other items that reduced the Cronbach’s alpha of the construct (i.e., “I am willing to spend an extra 10% for organic food”). We adopted this scale of price sensitivity because it has been developed and used in the Chinese context [36]. The items in this study included “It is acceptable to pay more for organic food than conventional food” and “I am willing to spend extra money in order to buy organic food.”

Organic food purchase intention was measured with three items adapted from Prakash et al. [17]. This scale measures consumers’ willingness and intention to purchase organic food in the near future. The items were “I am willing to buy organic food while shopping,” “I will make an effort to buy organic food in the near future,” and “I intend to buy organic products because they are more environmentally friendly.”

These measurement scales have been proved to have high reliability and validity in previous literature [17,35,36,37,40,44]. This study used a seven-point Likert type scale from one (strongly disagree) to seven (strongly agree) to measure all items.

### 3.4. Analysis Method

This study used SPSS 20 statistical software and structural equation model (SEM) with AMOS 18 to analyze the data and test the hypotheses. First, SPSS 20 was used to analyze descriptive statistics, correlation coefficients, and reliability of all variables. Second, SEM was used to conduct confirmatory factor analysis (CFA). Based on the results of this CFA model, convergent and discriminant validities were tested. Third, multiple regression analysis was used to test the hypotheses in this study. Finally, multiple regression analysis and a bootstrap method with SEM were used to test the moderated mediation effect of the hypothesized model.

## 4. Results

### 4.1. Descriptive Statistics and Correlation Analysis

Table 3 shows the descriptive statistics and correlation between variables. Results show that environmental consciousness was positively related to perceived food quality (*r* = 0.36, *p* < 0.01) and organic food purchase intention (*r* = 0.45, *p* < 0.01). Perceived food quality was positively related to organic food purchase intention (*r* = 0.45, *p* < 0.01). Furthermore, price sensitivity was positively related to organic food purchase intention (*r* = 0.42, *p* < 0.01).

### 4.2. Measurement Model

To test the model fit between the research model and empirical data, a CFA model was conducted with AMOS 18 statistical software. According to Kline [45], a good model fit is indicated when Chi-square/degree of freedom is less than 3, GFI (goodness of fit index), CFI (comparative fit index), and NFI (normed fit index) are all greater than 0.90, and RMSEA (root-mean-square error of approximation) is less than 0.08. The results of CFA in this study show that all criteria met these requirements (χ^2^/d.f. = 83.33/38 = 2.19, GFI = 0.97, CFI = 0.98, NFI = 0.97, and RMSEA = 0.05). Thus, this study shows a good model fit between empirical data and research models.

Internal consistency reliability is often tested using Cronbach’s alpha. Reliability is acceptable if the value of Cronbach’s alpha is greater than 0.70 [45,46]. Table 4 shows that Cronbach’s alpha for environmental consciousness, perceived food quality, price sensitivity, and organic food purchase intention were 0.82, 0.75, 0.70, and 0.90, respectively. Therefore, the reliability of all variables is satisfactory in this study.

This study followed Kline’s [45] suggestions and calculated composite reliability (CR), average variance extracted (AVE), and the square root of average variance extracted (√AVE) to test the validity of the measures. Table 4 shows the results of these indicators. Kline [45] noted that if CR is greater than 0.70 and AVE is greater than 0.50, then convergent validity is acceptable. Results in Table 4 indicate that all CR and AVE values of all variables exceed the thresholds. Thus, convergent validity is satisfactory in this study. Furthermore, Kline [45] suggested that when values of the square root of AVE of all variables are greater than all correlation coefficients, discriminant validity is acceptable. Results in Table 3 show that values of √AVE on the main diagonal exceed all correlation coefficients between variables. Thus, the discriminant validity of the measures in this study is satisfactory.

Podsakoff et al. [47] suggested that a self-reported survey should check the common method variance (CMV) problem due to the same respondent providing measures for independent and dependent variables simultaneously. Podsakoff et al. [47] stated that the CMV problem may occur if the results of the principal component analysis (PCA) with an unrotated solution show a single factor solution or the first factor accounts for a majority of the variance. Results of PCA in this study indicate that four factors emerged, which accounted for 76.54% of the variance. The first factor accounted for 35.16% of the variance. To confirm this result, a one-factor model of CFA was conducted in this study. Results of this CFA model present that all criteria did not meet the requirements of model fit (χ^2^/d.f. = 15.88, GFI = 0.79, CFI = 0.74, NFI = 0.73, and RMSEA = 0.17). This CMV problem may be ignored in this study.

### 4.3. Hypotheses Testing

This study used hierarchical regression approach to analyze data. Based on previous organic food literature [4,23,24], some common variables were controlled in the analysis due to their potential impact on a dependent variable (i.e., gender, age, marital status, income, and purchase experience). Table 5 presents the results of hierarchical regression analysis for testing the hypotheses in this study. As shown in Model 1a, purchase experience has a negative effect on organic food purchase intention (β = −0.13, *p* < 0.01). All other control variables were not significantly related to organic food purchase intention. Fundamentally, consumers’ intention to purchase organic food does not depend largely on their personal characteristics (i.e., gender, age, income, marriage).

Furthermore, Model 2a in Table 5 tests the direct effect of environmental consciousness on organic food purchase intention. Results show that environmental consciousness was positively related to organic food purchase intention (β = 0.54, *p* < 0.001). This result indicates that environmental consciousness enhances consumers’ intention to purchase organic food. Thus, H1 was supported.

This study followed the procedure proposed by Baron and Kenny [48] to test the mediation of perceived food quality in the link between environmental consciousness and organic food purchase intention. First, the independent variable must be significantly related to the dependent variable in the first model. Second, the independent variable must be significantly related to the mediating variable in the second model. Third, when regressing the independent and mediating variables on the dependent variable in the third model, the mediating variable must be significantly related to the dependent variable. If the effect of the independent variable on the dependent variable is significantly reduced, then partial mediation is supported. However, if the independent variable is insignificantly related to the dependent variable, then full mediation is supported. Accordingly, results from Models 2a–4a in Table 5 show that environmental consciousness was positively related to organic food purchase intention in Model 2a (β = 0.54, *p* < 0.001) and perceived food quality in Model 3a (β = 0.36, *p* < 0.001). Furthermore, perceived food quality was positively related to organic food purchase intention in Model 4a (β = 0.32, *p* < 0.001). Environmental consciousness was also positively related to organic food purchase intention in Model 4a (β = 0.42, *p* < 0.001). However, the effect of environmental consciousness on organic food purchase intention was significantly reduced in Model 4a (β = 0.42) as compared with that in Model 2a (β = 0.54). This result indicates that perceived food quality partially mediates the relationship between environmental consciousness and organic food purchase intention. This study also followed Preacher, Rucker, and Hayes [49] to conduct a bootstrap analysis with 1000 bootstrap samples to confirm the mediating effect of perceived food quality in the relationship between environmental consciousness and organic food purchase intention. Results show that the indirect effect of environmental consciousness on organic food purchase intention through perceived food quality was statistically significant (environmental consciousness perceived food quality organic food purchase intention: β = 0.073, *p* < 0.001, 95% CI = 0.031–0.140). This result provides further evidence for the mediating role of perceived food quality in the relationship between environmental consciousness and organic food purchase intention. Thus, H2 was supported.

**Figure 2:**
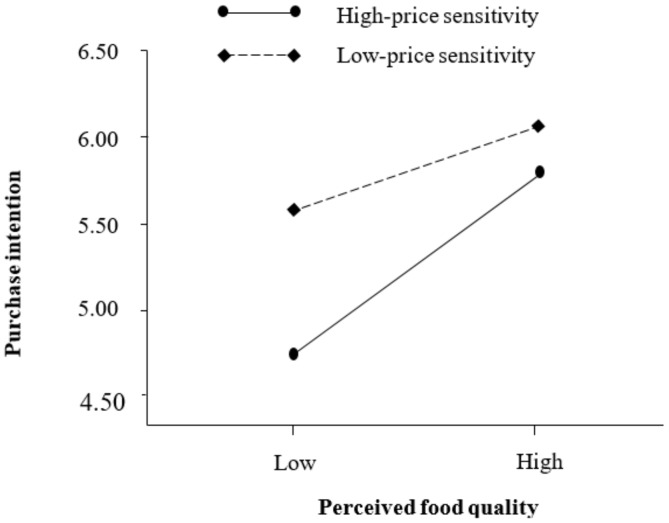
Moderating effect of price sensitivity.

This study adopted a multiple regression analysis method proposed by Baron and Kenny [48] to test the moderating role of price sensitivity on the relationship between perceived food quality and organic food purchase intention. Results are shown from Model 1b to 3b in Table 5. First, control variables were included in Model 1b. Second, the main effects of perceived food quality and price sensitivity on organic food purchase intention were tested in Model 2b. Third, the interaction effect between perceived food quality and price sensitivity on organic food purchase intention was tested in Model 3b. As indicated in Model 3b in Table 5 and Figure 2, the interaction effect between perceived food quality and price sensitivity was significantly and negatively related to organic food purchase intention (β = −0.11, *p* < 0.01). This result implies that price sensitivity weakens the relationship between perceived food quality and organic food purchase intention. Ultimately, the effect of perceived food quality on organic food purchase intention was stronger when price sensitivity is low and weaker when price sensitivity is high. Thus, H3 was supported.

This study also followed the procedure proposed by Edwards and Lambert [50] to conduct a bootstrap analysis for testing the moderated mediation hypothesis. Table 6 shows that the results of the second stage analysis reveal the effect of perceived food quality on organic food purchase intention for low- and high-price sensitivity groups. Results indicate that the influence of perceived food quality on organic food purchase intention varied significantly between the two groups (∆β = 0.071, *p* < 0.001). That is, the influence of perceived food quality on organic food purchase intention was stronger for low-price sensitivity group (β = 0.325, *p* < 0.001) than that for the high-price sensitivity group (β = 0.254, *p* < 0.001). This result provides further support for the moderating effect of price sensitivity on the link between perceived food quality and organic food purchase intention. Thus, H3 was further confirmed.

Furthermore, results in Table 6 also exhibit that the indirect effect of environmental consciousness on organic food purchase intention through perceived food quality is stronger when price sensitivity is low (β = 0.102, *p* < 0.001) and weaker when price sensitivity is high (β = 0.075, *p* < 0.001). The indirect effect of environmental consciousness on organic food purchase intention varied significantly between low and high levels of price sensitivity (∆β = 0.027, *p* < 0.01). Therefore, H4 was supported.

In sum, this study used analysis methods proposed by Baron and Kenny [48] and confirmed by analysis procedures proposed by Preacher et al. [49] and Edwards and Lambert [50] to test the mediation and moderation. The results of these methods provide evidence to support H2, H3, and H4. That is, the mediating roles of perceived food quality and price sensitivity were supported in this study.

## 5. Discussion and Implications

This study aims to investigate the relationship between environmental consciousness and organic food purchase intention with the mediating roles of perceived food quality and price sensitivity. This study finds several interesting results. Environmental consciousness has a positive influence on organic food purchase intention. This finding suggests that consumers care more about environmental issues and that they integrate environmental impacts into their purchase decisions [19,32]. Environmentally conscious consumers may think that if they purchase more green products, then their purchase behavior will motivate business firms to invest more effort and resources to produce environmentally friendly products and protect the environment [19,22]. Fundamentally, when consumers care about environmental issues, they tend to reduce their impact on the environment. This belief motivates consumers to purchase organic food products because they suppose that organic food has less environmental impact than conventional food. Our findings support the research of Mishal et al. [1] and D’Amico et al. [31], which found that environmentally conscious consumers tend to care about environmental problems and purchase more environmentally friendly products.

Furthermore, perceived food quality has a mediating effect on the link between environmental consciousness and organic food purchase intention. This finding indicates that when consumers care about the environment, they will hold perceptions about the impact of food products on the environment. That is, consumers link the quality of organic food with the environment and decide their purchase decision [3,22,31]. When considering the environmental impact of food products, consumers may perceive and evaluate organic food as of high quality because organic food is generally known as natural products without chemicals. Organic food possesses a higher quality than conventional food because organic food is nutritious, fresher, and safer. Hence, consumers will purchase more organic food products [30,31]. Our findings support Leaniz et al. [2], Chekima et al. [4], and Scalco et al. [26] who suggested that consumers who often purchase green products do so because they believe that green products have higher quality and less negative impact on the environment.

In addition, price sensitivity moderates the relationship between perceived food quality and organic food purchase intention. Price sensitivity also moderates the indirect effect of environmental consciousness on organic food purchase intention through perceived food quality. These findings suggest that consumers who are price-sensitive may take price as a key consideration in their perceptions and behavioral intentions [4,19,24]. When the price is high, consumers’ perceptions about environmental issues, quality of products, and their purchase intention will be substantially influenced because they view price as a key factor in their decision-making process. By contrast, perceptions and decisions of consumers who care less about price are not likely to be affected by the price. These consumers will care more about the environment and quality of organic products, and they will purchase more organic products [19,37,38]. Our findings support Prakash et al. [17] and Rana and Paul [38] who reported that price plays an important role in consumers’ decision-making when they purchase environmentally friendly products.

### 5.1. Research Implications

First, this study sheds new light on the relationship between environmental consciousness and organic food purchase intention. In the last few years, researchers and business managers focus on the environmental issue [44]. As consumers care more about the environment, they demand more environmentally friendly products [1]. The market for organic food also increases rapidly because organic food is viewed as healthy products that generate several benefits for human health [10]. This study determines the impact of environmental consciousness on purchase intention to understand consumers’ perceptions and behavioral intentions toward organic food. Our findings indicate that people who care about the environment often consider the impact of their purchase behavior on the environment [19]. Therefore, they tend to purchase organic food because they may perceive that organic food is safer and healthier and has less impact on the environment and ecological system [22]. The findings of this study provide evidence to support previous studies [2,30,31] which suggested that environmental concern is associated with consumers’ intention to purchase green products.

Second, the findings of this study also clarify the mediating mechanism of perceived food quality in the link between environmental consciousness and purchase intention. Our findings suggest that when consumers care about the environment, they tend to evaluate food products with regard to the environment [1]. As compared with conventional food, consumers often evaluate organic food as of higher quality because it is naturally produced without chemicals and pesticides [31]. Hence, consumers will purchase more organic food because they perceive that organic food is of high quality and has less impact on the environment [3,22]. The findings of this study enrich empirical evidence on consumers’ perception and behavioral intention toward organic food.

Third, this study also helps to clarify the moderating mechanism of price sensitivity on the relationship between perceived food quality and purchase intention. Although price is often a key factor in consumers’ decision-making, this notion may differ between consumers when they purchase organic food [24]. Consumers who care about price may be very sensitive to the price of organic food. When the price of organic food is high, these consumers may hold low perceptions about the quality and purchase less organic food. Instead, these consumers may choose conventional food as a substitute for organic food [38]. By contrast, consumers who are price-insensitive may place more consideration on nonprice factors (i.e., quality) of organic food [19]. When organic food is perceived as of higher quality than conventional food, these consumers may purchase more organic food [4]. Furthermore, this study also finds that price sensitivity also moderates the indirect effect of environmental consciousness on purchase intention through perceived food quality. This finding provides further evidence of the effect of price on consumers who are price sensitive versus those who are price-insensitive [37]. Therefore, this study extends previous organic food literature by providing empirical evidence on the moderating mechanism in the relationship between environmental consciousness and purchase intention.

Ultimately, China has become the largest economy in emerging markets. The country’s economy has grown rapidly in recent decades. However, the development of industrialization has damaged the environment and entailed several social problems [18]. As Chinese consumers become more powerful in their income and knowledgeable in their purchase decisions, they care more about environmental issues, social welfare, and health conditions. Consequently, Chinese consumers demand more environmentally friendly products and organic food [19]. Business managers must understand consumers’ perceptions and behaviors toward organic food to construct better decisions and satisfy Chinese consumer needs. Therefore, this study contributes to researchers in China who may be interested in studying the issue of organic food in the Chinese market. Given that China has faced several environmental and ecological problems in recent decades, the market for organic food in China is increasing rapidly. Knowledge about organic food will help researchers understand better consumers’ perceptions and behaviors in the food industry in China. The findings of this study will provide implications for managers of domestic and foreign food companies to make better decisions and policies in China’s food market.

### 5.2. Practical Implications

This study also provides several implications for retailer managers. On the basis of the empirical findings of this study, retailer managers in the food industry and food companies should plan and execute marketing strategies to communicate and persuade consumers to purchase more organic food. For example, retailer managers should use advertising campaigns to link organic food with environmental issues. Retailer managers can persuade consumers to purchase organic food by emphasizing organic food as environmentally friendly products without any harmful elements to human health and the environment. Furthermore, retailer managers should also use different marketing campaigns to communicate value with consumers. Managers can raise consumers’ perceptions about the difference between organic and conventional foods by positioning as high-value products and conveying high-quality organic food. In addition, retailer managers should also design different product combinations to satisfy various consumers. For price-sensitive consumers, a suitable quality with acceptable price organic food should be provided for this target market. By contrast, organic food of high quality with high price should be provided for consumers who do not care about the price. A good marketing strategy will change consumers’ perceptions and behaviors toward organic food products.

## 6. Conclusions and Future Research

This study helps to clarify the influence of environmental consciousness on organic food purchase intention with the mediating role of perceived food quality and the moderating role of price sensitivity. The findings of this study provide implications for researchers and business managers regarding the management and development of organic food. Specifically, our findings advance the knowledge of the field and help business managers plan and execute strategies for China’s organic food market. However, this study has also several limitations. This study used a questionnaire survey to collect data. Given that the same respondents provide measures for all variables simultaneously, common method bias may affect the results. Thus, future research should overcome this limitation by collecting data from the same respondents in a different time period. Furthermore, cross-sectional data may affect the results of the analysis, and the causal relationship between variables is better tested with longitudinal data. Future research should collect longitudinal data to validate the causal relationships in this study. In addition, our data were collected from consumers in different food retailers in China. The generalization of the results may be a limitation. Therefore, future research should collect data in different countries to determine organic food issues. Finally, many variables may play a mediating and/or moderating effect in the relationship between antecedents and purchase behavior of organic food. Future research should consider more new variables to clarify the impact of consumers’ psychological factors on purchase behavior.

## Figures and Tables

**Table 1:** Demographics of respondents.

Variable	Frequency	Percent
Gender		
*Male*	186	35.90%
*Female*	332	64.10%
Age		
*25 or below*	37	7.10%
*26*–*35*	268	51.70%
*36*–*45*	175	33.80%
*46*–*55*	28	5.40%
*56 or above*	10	1.90%
Marital status		
*Married*	501	96.70%
*Not married*	17	3.30%
Education		
*High school or below*	352	68.00%
*University or college*	152	29.30%
*Graduate or above*	14	2.70%
Income		
*Under 3000 RMB*	136	26.30%
*3000*–*under 6000 RMB*	226	43.60%
*6000*–*under 9000 RMB*	96	18.50%
*9000*–*under 12,000 RMB*	41	7.90%
*12,000 RMB or above*	19	3.70%
Whether or not previously purchased organic food		
*Yes*	418	80.70%
*No*	100	19.30%

Note: *n* = 518.

**Table 2:** Constructs and items.

Constructs	Items	Sources
Environmental consciousness	The balance of nature is very delicate and can be easily upset. I have switched products for ecological reasons. When I have a choice between two equal products. I purchase the one less harmful to other people and the environment.	[17]
Perceived food quality	There is a high likelihood that organic food bought at this store will be of extremely high quality. When shopping at this store, I expect to see high quality organic food. Overall, this store sells high quality organic food.	[40]
Price sensitivity	It is acceptable to pay more for organic food than conventional food. I am willing to spend extra money in order to buy organic food.	[36]
Organic food purchase intention	I am willing to buy organic food while shopping. I will make an effort to buy organic food in the near future. I intend to buy organic products because they are more environmentally friendly.	[17]

**Table 3:** Means, standard deviations, and Pearson correlations.

Variable	Mean	S.D.	1	2	3	4
1. Environmental consciousness	5.64	1.00	0.78			
2. Perceived food quality	5.41	0.97	0.36 ^**^	0.77		
3. Price sensitivity	5.11	1.02	0.31 ^**^	0.30 ^**^	0.72	
4. Organic food purchase intention	5.48	0.96	0.45 ^**^	0.47 ^**^	0.42 ^**^	0.86

Note: *n* = 518, ** *p* < 0.01, diagonal values are square roots of average variance extracted (AVE).

**Table 4:** Confirmatory factor analysis results.

Constructs	Items	Loadings	CR	AVE	√AVE	Cronbach’s α
Environmental consciousness	ENC1	0.76	0.83	0.61	0.78	0.82
	ENC2	0.85				
	ENC3	0.74				
Perceived food quality	PFQ1	0.66	0.74	0.59	0.77	0.75
	PFQ2	0.87				
	PFQ3	0.73				
Price sensitivity	PRP1	0.82	0.76	0.52	0.72	0.70
	PRP2	0.60				
Organic food purchase intention	OFP1	0.88	0.90	0.74	0.86	0.90
	OFP2	0.85				
	OFP3	0.87				

Note: *n* = 518. CR = composite reliability, AVE = average variance extracted, √AVE = square root of AVE.

**Table 5:** Results of the hypothesis testing.

Variable	Model 1a (Purchase Intention)	Model 2a (Purchase Intention)	Model 3a (Perceived Quality)	Model 4a (Purchase Intention)	Model 1b (Purchase Intention)	Model 2b (Purchase Intention)	Model 3b (Purchase Intention)
Control variable							
*Gender*	0.53	0.42	0.04	0.03	0.53	0.02	0.02
*Age*	0.01	−0.01	−0.01	0.01	0.01	0.01	0.01
*Marital status*	−0.02	−0.01	0.05	−0.03	−0.02	−0.05	−0.04
*Income*	0.06	0.88	0.01	0.03	0.06	0.05	0.04
*Purchased experience*	−0.13 ^**^	−0.08 ^*^	−0.08	−0.06	−0.13 ^**^	−0.05	−0.06
Independent variable							
*Environmental consciousness*		0.54 ^***^	0.36 ^***^	0.42 ^***^			
Mediator							
*Perceived food quality*				0.32 ^***^		0.38 ^***^	0.38 ^***^
Moderator							
*Price sensitivity*						0.29 ^***^	0.31 ^***^
Interaction							
*Perceived food quality × Price sensitivity*							−0.11 ^**^
F value	2.368 ^*^	32.934 ^***^	12.122 ^***^	41.990 ^***^	2.368 ^*^	30.298 ^***^	28.269 ^***^
∆F		30.566 ^***^	9.754 ^***^	39.622 ^***^		27.930 ^***^	2.029 ^**^
R square	0.027	0.311	0.143	0.398	0.027	0.323	0.334
∆R square		0.284	0.116	0.371		0.296	0.011

Note: *n* = 518, * *p* < 0.05, ** *p* < 0.01, *** *p* < 0.001.

**Table 6:** Results of the moderated mediation path analysis.

Moderator	Environmental Consciousness (X) → Perceived Food Quality (M) → Organic Food Purchase Intention (Y)				
	Stage		Effect		
	First (P_XM_)	Second (P_MY_)	Direct Effects (P_XY_)	Indirect Effects (P_XM_P_MY_)	Total Effects (P_XY_ + P_XM_P_MY_)
Low-price sensitivity	0.313 ^***^	0.325 ^***^	0.411 ^***^	0.102 ^***^	0.513 ^***^
High-price sensitivity	0.297 ^***^	0.254 ^**^	0.394 ^***^	0.075 ^***^	0.469 ^***^
Differences	0.016 ^***^	0.071 ^***^	0.017 ^*^	0.027 ^**^	0.044 ^**^

Note: *n* = 518, * *p* < 0.05, ** *p* < 0.01, *** *p* < 0.001; P_XM_: path from independent variable to mediator; P_MY_: path from mediator to dependent variable; P_XY_: path from independent variable to dependent variable.
